# CSF-Neurofilament Light Chain Levels in NMDAR and LGI1 Encephalitis: A National Cohort Study

**DOI:** 10.3389/fimmu.2021.719432

**Published:** 2021-12-16

**Authors:** Mette Scheller Nissen, Matias Ryding, Anna Christine Nilsson, Jonna Skov Madsen, Dorte Aalund Olsen, Ulrich Halekoh, Magnus Lydolph, Zsolt Illes, Morten Blaabjerg

**Affiliations:** ^1^ Department of Neurology, Odense University Hospital, Odense, Denmark; ^2^ Department of Clinical Research, University of Southern Denmark, Odense, Denmark; ^3^ Brain Research - Inter Disciplinary Guided Excellence (BRIDGE), Odense, Denmark; ^4^ Department of Neurobiology Research, Institute of Molecular Medicine, University of Southern Denmark, Odense, Denmark; ^5^ Department of Clinical Immunology, Odense University Hospital, Odense, Denmark; ^6^ Department of Biochemistry and Immunology, Lillebælt Hospital, University Hospital of Southern Denmark, Vejle, Denmark; ^7^ Department of Regional Health Research, University of Southern Denmark, Odense, Denmark; ^8^ Department of Epidemiology, Biostatistics and Biodemography, Institute of Public Health, University of Southern Denmark, Odense, Denmark; ^9^ Danish National Biobank, Statens Serum Institut, Copenhagen, Denmark

**Keywords:** autoimmune encephalitis (AE), neurofilament light (NfL) chain, biomarker, NMDAR encephalitis (NMDARE), LGI1 encephalitis, outcome

## Abstract

**Background and Objectives:**

The two most common autoimmune encephalitides (AE), *N*-methyl-D-Aspartate receptor (NMDAR) and Leucine-rich Glioma-Inactivated 1 (LGI1) encephalitis, have been known for more than a decade. Nevertheless, no well-established biomarkers to guide treatment or estimate prognosis exist. Neurofilament light chain (NfL) has become an unspecific screening marker of axonal damage in CNS diseases, and has proven useful as a diagnostic and disease activity marker in neuroinflammatory diseases. Only limited reports on NfL in AE exist. We investigated NfL levels at diagnosis and follow-up in NMDAR and LGI1-AE patients, and evaluated the utility of CSF-NfL as a biomarker in AE.

**Methods:**

Patients were included from the National Danish AE cohort (2009-present) and diagnosed based upon autoantibody positivity and diagnostic consensus criteria. CSF-NfL was analyzed by single molecule array technology. Clinical and diagnostic information was retrospectively evaluated and related to NfL levels at baseline and follow-up. NMDAR-AE patients were subdivided into: idiopathic/teratoma associated or secondary NMDAR-AE (post-viral or concomitant with malignancies/demyelinating disease).

**Results:**

A total of 74 CSF samples from 53 AE patients (37 NMDAR and 16 LGI1 positive) were included in the study. Longitudinal CSF-NfL levels was measured in 21 patients. Median follow-up time was 23.8 and 43.9 months for NMDAR and LGI1-AE respectively. Major findings of this study are: i) CSF-NfL levels were higher in LGI1-AE than in idiopathic/teratoma associated NMDAR-AE at diagnosis; ii) CSF-NfL levels in NMDAR-AE patients distinguished idiopathic/teratoma cases from cases with other underlying etiologies (post-viral or malignancies/demyelinating diseases) and iii) Elevated CSF-NfL at diagnosis seems to be associated with worse long-term disease outcomes in both NMDAR and LGI1-AE.

**Discussion:**

CSF-NfL measurement may be beneficial as a prognostic biomarker in NMDAR and LGI1-AE, and high CSF-NfL could foster search for underlying etiologies in NMDAR-AE. Further studies on larger cohorts, using standardized methods, are warranted.

## Introduction

The two most common types of autoimmune encephalitides (AE) are *N*-methyl-D-aspartate receptor encephalitis (NMDAR-AE) and Leucine-rich Glioma-Inactivated 1 encephalitis (LGI1-AE) (1–3). LGI1-AE commonly affects middle-aged and older adults, has a male predisposition and presents with seizures and cognitive decline. NMDAR-AE mainly affects children and young adults, predominantly females and presents with psychiatric symptoms, seizures, decreased consciousness and autonomic dysfunction (4–7). While LGI1-AE is mostly thought to be a non-complement fixing Immunoglobin G4 (IgG4) mediated disease, NMDAR-AE is driven mainly by IgG1, which has the potential to initiate complement activation (8–10). NMDAR-AE can be seen in combination with other diseases, such as malignancies, demyelinating diseases or after herpes simplex virus 1 encephalitis (HSE) (4, 11–13).

AE’s can be challenging to treat, and good biomarkers to assist physician’s decision-making are scarce. Naturally, immunological biomarkers have been in focus in AE patients. Thus, Chemokine (C-X-C motif) ligand 13 (CXCL-13) has been reported as a potential biomarker of treatment response and relapse rate in a bigger cohort of NMDAR-AE patients, but long-term outcomes in relation to CXCL-13 remains to be further elucidated (14). Similarly, cytokine studies, mostly in NMDAR-AE patients, have suggested particularly Interleukin-17A as a biomarker of disease severity at onset and a poor prognosis (15–18). Limited studies have investigated neuronal surface autoantibody titers as diagnostic or prognostic biomarkers and high titers may indicate poor prognosis and malignancy (4, 19, 20). Studies with acute and longitudinal measurements of autoantibodies and clinical correlates are limited, and patients can display high titers years after recovery (8, 21). The knowledge on biomarkers of neuronal damage in relation to long-term outcomes in AE remains scarce.

Neurofilament light chain (NfL) are scaffolding proteins solely expressed in the neuronal cytoskeleton and a reliable indicator of axonal damage. They have been studied as emerging biomarkers of neurodegeneration, but also increasingly as a biomarker in neuroinflammation-related axonal damage. NfL has been shown to correlate with disease severity and progression/outcome in diseases like optic neuritis, multiple sclerosis, neurosarcoidosis and myelin oligodendrocyte glycoprotein associated diseases (22–27).

Here we investigate CSF-NfL levels in the acute phase of NMDAR- and LGI1-AE patients and evaluate its utility as a diagnostic and prognostic biomarker in the two commonest types of AE.

## Material and Methods

### Patient Materials and Samples

From 2009-2018, 74 CSF samples (49 NMDAR and 25 LGI1 IgG positive) from 53 patients (37 NMDAR-AE and 16 LGI1-AE) were obtained from the two only test centers in Denmark (Danish National Biobank, Statens Serum Institut, Copenhagen and Department of Clinical Immunology, Odense University Hospital). Medical charts and diagnostic work-up were retrospectively evaluated using the consensus criteria for NMDAR-AE and autoimmune limbic encephalitis (LE) (28). Thus, all patients included in this study (regardless of concomitant disease or underlying malignancies) fulfilled criteria for AE (except one patient with LGI1 IgG and faciobrachial dystonic seizures (FBDS) only) and displayed a clinical phenotype compatible with AE.

Antibody positivity was reported qualitatively as weakly (+), moderately (++) and strongly positive (+++) and based on a commercial cell-based assay (CBA, Euroimmun AG, Lübeck, Germany). Abnormal CSF was defined as pleocytosis (>5 cells/μL), elevated protein levels and/or presence of CSF specific oligoclonal bands (OCB). An abnormal MRI was defined as T2/FLAIR hyperintensities in NMDAR-AE and temporal lobe T2/FLAIR hyperintensities in LGI1-AE. The modified Rankin Scale (mRS) was used to determine neurological disability at last follow-up. A mRS score > 2 was considered a poor outcome and last follow-up was the last obtainable clinical neurological assessment. The CSF sample NfL was measured on, was obtained within one month of diagnosis, and prior to any treatment in all patients.

### CSF Neurofilament Light Assay

CSF-NfL was measured blinded to clinical data by single molecule array technology (Simoa, HD-X Analyzer (Quanterix)), using a commercial NfL assay (Quanterix, Billerica, MA, USA). CSF samples used were routine samples from patient’s initial hospital stay, stored at -80°C. Quality control was performed using two commercial controls provided by the manufacturer. In addition, an in-house CSF pool, from routine CSF samples, was used as internal control. Lower limit of detection and quantification was 0.038 pg/mL and 0.174 pg/mL, respectively. Age adjusted CSF-NfL values were determined according to a previous study and applied when performing univariable analysis (29). Thus, elevated CSF-NfL levels were as follows: 18-40 years <560 pg/mL; 40-60 years <890 pg/mL and > 60 years <1850 pg/mL. These reference values are in line with what was found in >1300 healthy controls and >2700 patients with inflammatory CNS diseases in a large systematic review and meta-analysis (30).

### Ethical Approval

Ethical approval was obtained by the Danish Medical Authorities (3-3013-3124/1) and the Danish Data Protection Agency, with waived requirement for participant consent.

### Statistical Analysis

Statistical analysis was performed using GraphPad Prism (version 6.0.0 for macOS, GraphPad software, San Diego, California, USA) and stataCorp. 2021 (*Stata Statistical Software: Release 17*. College Station, TX: StataCorp LLC). NfL data were skewed and thus natural logarithmic transformed. Post-HSE and secondary NMDAR-AE forms were excluded from all stastistical analyses (except **Figure 2C**), as concomitant disease and prior HSE might interfere with NfL values. Single factors potentially influencing outcome and NfL were analyzed by unpaired t-test. Multivariable analysis of outcome was performed on idiopathic/teratoma associated NMDAR-AE and LGI1-AE patients combined to achieve sufficient sample size for analysis. Parameters analyzed for an effect on poor outcome (mRS>2) were logNfL, age and follow-up time (all continuous). Parameters analyzed for an effect on NfL (elevated or not) were age (continuous), pleocytosis (continuous) and abnormal MRI (binary). A p-value ≤ 0.05 was considered statistically significant.

## Results

### Patient Demographics, Phenotypes and Ancillary Testing

A total of 53 AE patients (37 NMDAR-AE and 16 LGI1-AE) were included in this study. Twenty-three (62%) NMDAR-AE patients were female with a median age of 27 years (**Table 1**). CSF pleocytosis was present in 33 (89%) and an abnormal brain MRI was found in 20 patients (54%) (**Table 1**). Seven (44%) LGI1-AE patients were female and median age at onset was 63 years (**Table 1**). All LGI1-AE patients except one displayed a classic LE; one had isolated FBDS. Only one LGI1-AE patient had CSF pleocytosis and 10 (59%) displayed an abnormal brain MRI (**Table 1**). The median follow-up time was 23.8 months (725 days) and 43.9 months (1337 days) for NMDAR-AE and LGI1-AE patients, respectively (**Table 1**). First NfL measurement was within 1 months of diagnosis and before treatment initiation in all patients. An overview of each patient is provided as supplementary material (**Supplementary Table**).

**Table 1:** Demographics, diagnostic information and outcomes of the AE cohort.

AE (n = 53)					
	LGI1-AE (n = 16)	NMDAR-AE (n = 37)			
		All (n = 37)	Idiopathic/teratoma (n = 27)	Secondary post-HSE (n = 5)	Secondary Other (n = 5)^a^
**Demographics**					
Sex, female	7 (44%)	23 (62%)	20 (74%)	2 (40%)	1 (20%)
Age, median (range), years	63 (30-82)	27 (11-74)	21 (11-65)	65 (46-70)	56 (30-74)
**Diagnostic information**					
Fulfilling diagnostic criteria (NMDAR-AE)	–	35 definite/2 probable	27 definite	4 definite/1 probable	4 definite/1 probable
Limbic encephalitis (LGI1-AE)	15 LE/1 with FBDS only	–	–	–	–
CSF pleocytosis	1 (7%)^b^	33 (89%)	25 (93%)	5 (100%)	3 (60%)
Abnormal MRI during acute phase	10 (59%)	20 (54%)	12 (44%)	3 (60%)	5 (100%)
CSF NfL-level at treatment initiation, median (range)	1178.5 pg/mL (395-4722)	429 pg/mL (34-28048)	284 pg/mL (34-1812)	12409 pg/mL (4898-22458)	2285 pg/mL (1969-28048)
Elevated CSF NfL at treatment initiation	8 (50%)	15 (41%)	5 (19%)	5 (100%)	5 (100%)
Time from first symptom to diagnosis, days, median (range)	113.5 (31-440)	–	38 (5-176)^c^	–	–
**Treatment**					
Time from first symptom to treatment, days, median (range)	111 (10-440)	–	28.5 (4-170)	–	–
**Outcome**					
Follow-up time, months, median (range)	43.9 (1.15-86.31)^c^	23.8 (0.16-78.32)^d^	26 (0.16-78.32)^d^	22.8 (10.81-40.97)	17.3 (6.73-62.88)
mRS at follow-up, mean (range 1-6)	1,62	1,92	1,26*	3	4,4

a This group consisted of: one patient with SCLC and LE with anti-hu abs, one patient with PCNSL (B-cell), two patients with MS, one patient with ADEM.

b One patient NA.

c Two patients NA.

d Three patients NA.

AE, Autoimmune Encephalitis; LGI1, Leucine-rich Glioma-Inactivated 1; NMDAR, N-methyl-D-aspartate receptor; Post-HSE , Post Herpes Simplex Virus type 1 encephalitis; CSF, cerebrospinal fluid; MRI; Magnetic Resonance Imaging; NfL, Neurofilament Light Chain; mRS, Modified Rankin scale; SCLC, Small-cell lung cancer; LE, Limbic encephalitis; PCNSL, Primary CNS lymphoma; MS, multiple sclerosis; ADEM, Acute disseminated encephalomyelitis; NA, Not Available

### Subdivision of NMDAR-AE Patients Based on Underlying Etiology

All NMDAR-AE patients had a classic phenotype with a variety of changed behavior, memory impairment, psychiatric symptoms, insomnia, reduced verbal output, decreased consciousness and dysautonomia. Five patients had underlying tumors (three benign teratomas, one primary CNS B-cell lymphoma and one small-cell lung cancer). Three patients had concomitant demyelinating diseases; two multiple sclerosis and one acute disseminated encephalomyelitis. The three patients with teratomas showed a similar phenotype and disease course to idiopathic NMDARE patients, while patients with malignancies and demyelinating diseases showed a more extensive clinical syndrome than regular AE. NMDAR-AE patients were therefor subdivided into three groups: idiopathic/teratoma associated, secondary post-HSE and secondary other (concurrent malignancies or demyelinating disease) (**Table 1** and **Supplementary Table**). Secondary NMDAR-AE patients were more often male, middle-aged, more frequently had an abnormal MRI and had a worse outcome at final follow-up, when compared to idiopathic/teratoma associated NMDAR-AE and LGI1-AE patients (**Table 1**).

### CSF-NfL Levels at Diagnosis Are Higher in LGI1-AE Than in Idiopathic/Teratoma Associated NMDAR-AE

**Figure 1:**
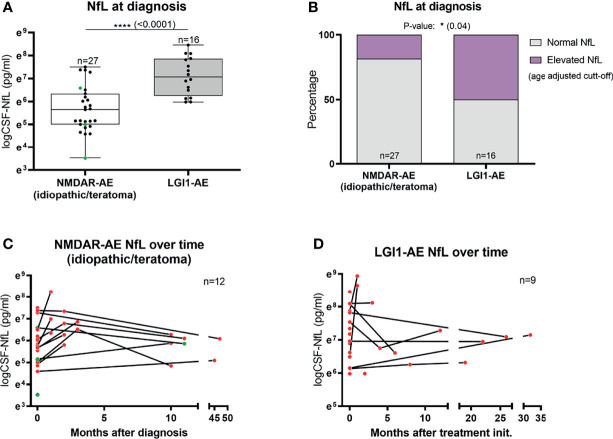
CSF-Neurofilament light chain (NfL) levels at diagnosis and follow-up. CSF-NfL levels at diagnosis were higher in LGI1 patients than in patients with idiopathic/teratoma associated NMDAR-AE **(A)**. In addition, LGI1-AE patients more often presented elevated NfL levels at diagnosis, when using age-adjusted cut-off levels of CSF-NfL **(B)**. Twelve NMDAR-AE (out of 27) **(C)** and nine LGI1-AE patients (out of 16) **(D)** had follow-up NfL measurements available. Idiopathic/teratoma associated NMDAR-AE patients CSF-NfL levels decreased over time in majority of cases **(C)**. In contrast, LGI1-AE patients CSF-NfL levels were more heterogenous during follow-up **(D)**. Idiopathic/teratoma associated NMDAR-AE patients with teratomas are shown as green datapoints **(A, C)**. *p value < 0.05, ****p value < 0.0001.

The median CSF-NfL concentration at diagnosis in LGI1-AE patients (n=16) was higher than in idiopathic/teratoma associated NMDAR-AE (n=27) (1178.5 pg/mL vs 284 pg/mL, p<0.0001) (**Table 1** and **Figure 1A**). Applying age adjusted cut-off values for CSF-NfL elevation revealed that only five idiopathic/teratoma associated NMDAR-AE patients (19%) had elevated CSF-NfL at diagnosis, compared to 50% of LGI1-AE patients (p=0.04) (**Table 1** and **Figure 1B**).

### CSF-NfL Kinetics Over Time

Twelve idiopathic/teratoma associated NMDAR-AE and nine LGI1-AE patients had CSF available for NfL measurement during follow-up (**Figures 1C, D**). Median time between initial and follow-up CSF-NfL measurement was 2.96 months (range 0.99-45.34 months) in idiopathic/teratoma NMDAR-AE and 7.88 months (range 0.26-48.30 months) in LGI1-AE patients. CSF-NfL levels declined in the majority of idiopathic/teratoma associated NMDAR-AE patients within 3 to 9 months after diagnosis, whereas CSF-NfL levels during follow-up in LGI1-AE patients were more heterogenous (**Figures 1C, D**).

### CSF-NfL Level at Diagnosis May Be Associated With a Poorer Outcome

**Figure 2:**
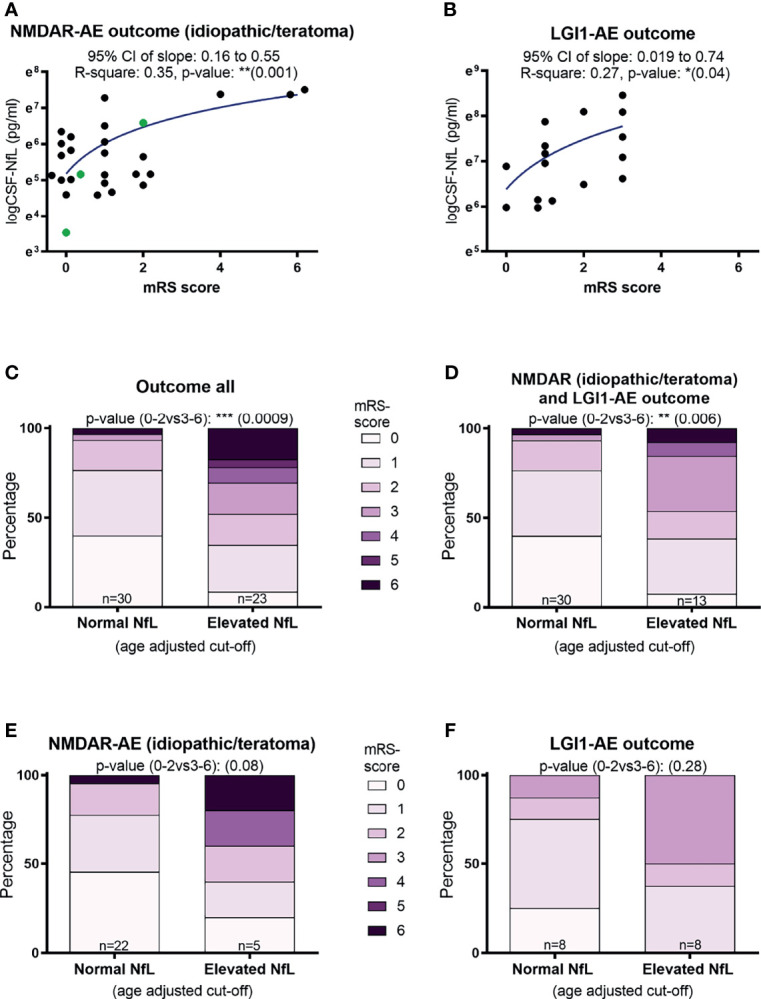
CSF-Neurofilament light chain (NfL) levels at diagnosis and patient outcomes. Higher CSF-NfL values at diagnosis was associated with worse outcome at last follow-up in both NMDAR and LGI1-AE patients **(A, B)**. Applying age-adjusted cut-off values for elevated CSF-NfL, showed that elevated NfL at diagnosis was associated with a poorer outcome in both the complete cohort tested and in idiopathic/teratoma associated NMDAR- and LGI1-AE patients combined **(C, D)**. When further dividing the cohort into idiopathic/teratoma associated NMDAR-AE patients **(E)** and LGI1-AE patients alone **(F)**, patients with elevated NfL at diagnosis still showed a poorer outcome, but no longer significant. NMDAR-AE patients with teratomas are shown as green datapoints **(A)**. *p value < 0.05, **p value < 0.01, ***p value < 0.001.

A higher CSF-NfL level at diagnosis was associated with a higher mRS score at final follow-up in both idiopathic/teratoma associated NMDAR-AE and LGI1-AE (p=0.001 and p=0.04) (**Figures 2A, B**). When applying age related cut offs for CSF-NfL and evaluating the entire AE cohort and the idiopathic/teratoma associated NMDAR- and LGI1-AE combined, an association between elevated CSF-NfL at diagnosis and a poor outcome was found (p=0.0009 and p=0.006) (**Figures 2C, D**). Examining idiopathic/teratoma associated NMDAR- and LGI1-AE patients separately, there was no significant association between CSF-NfL and poor outcome (**Figures 2E, F**). Nevertheless, patients with elevated NfL still scored higher mRS at follow-up in both subgroups. Thus, patients with an elevated CSF-NfL at diagnosis might be at greater risk of a poorer outcome. Distribution of outcome in each group of the entire cohort can be found in **Supplementary Figure**.

### CSF-NfL Levels Seem to Distinguish Idiopathic/Teratoma Associated NMDAR-AE From NMDAR-AE Secondary to Other Diseases

**Figure 3:**
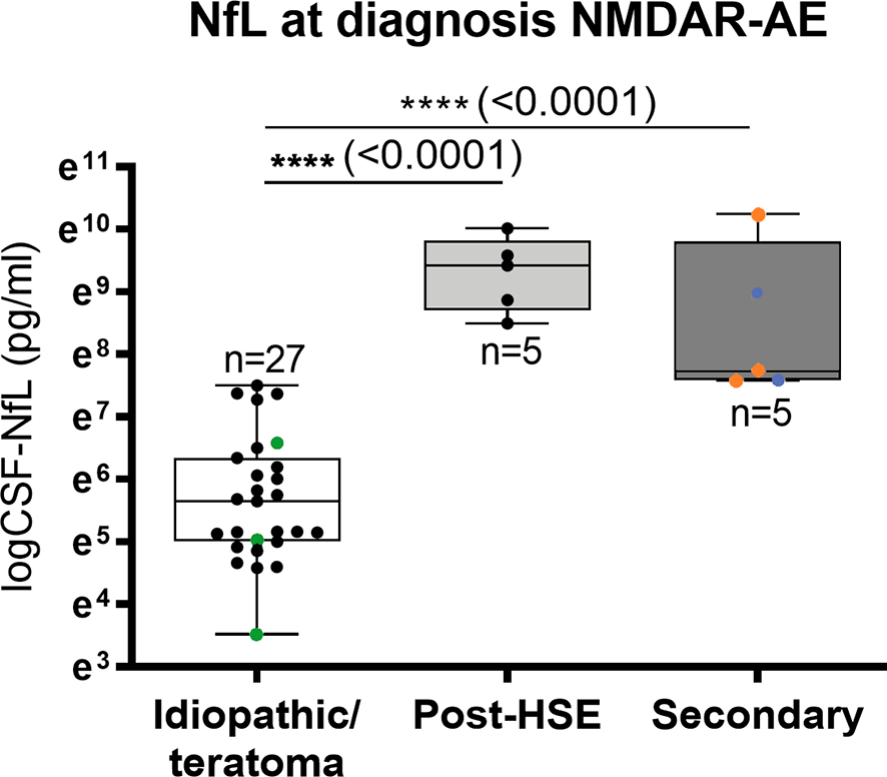
CSF-Neurofilament light chain (NfL) levels in NMDAR-AE subgroups. Patients with idiopathic/teratoma associated NMDAR-AE had significantly lower CSF-NfL levels at diagnosis than patients with secondary NMDAR-AE due to post-Herpes Simplex Virus 1 Encephalitis (Post-HSE) or underlying demyelinating conditions or malignancies (secondary). NMDAR-AE patients with teratomas are shown as green datapoints, demyelinating as red and malignancies as blue datapoints. ****p value < 0.0001.

CSF-NfL analysis showed different median concentrations at diagnosis amongst the NMDAR-AE subgroups (**Table 1** and **Figure 3**). Idiopathic/teratoma associated NMDAR-AE patients had lower CSF-NfL levels compared to both post-HSE and secondary NMDAR-AE patients (median 284 vs 12409 pg/mL (p<0.0001) and 2285 pg/mL (p<0.0001)) (**Table 1** and **Figure 3**). All secondary NMDAR-AE patients (both post HSE and with concomitant diseases) had elevated NfL levels at diagnosis.

### CSF-NfL Levels and Antibody Positivity in AE

All NMDAR-AE and 14/15 (one sample was unavailable) of the LGI1-AE patients had autoantibodies present in the CSF at diagnosis (**Supplementary Table**). In idiopathic/teratoma associated NMDAR-AE patients, increasing intensity in antibody positivity in serum associated with higher CSF-NfL levels (- vs +, p=0.03 and - vs ++, p=0.03) (data not shown). Otherwise, no associations were seen in either idiopathic/teratoma associated NMDAR-AE in CSF or in LGI1-AE in serum or CSF.

### Clinical Phenotype, Paraclinical Findings and CSF-NfL Levels

**Figure 4:**
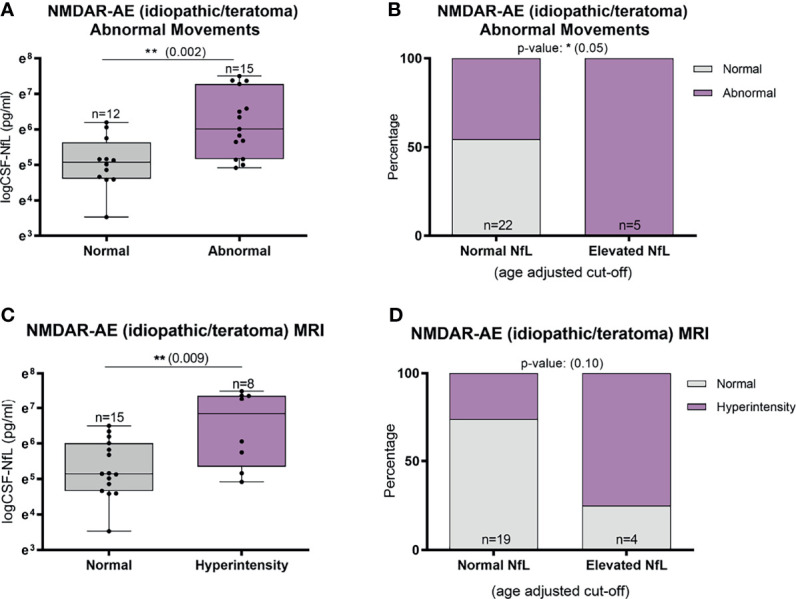
CSF-Neurofilament light chain (NfL) levels in relation to abnormal involuntary movements and MRI findings in idiopathic/teratoma associated NMDAR-AE patients. Patients with NMDAR-AE (idiopathic or teratoma) and abnormal movements had higher NfL levels at diagnosis **(A)**, and all patients with elevated NfL levels presented abnormal movements **(B)**. MRI hyperintensities on T2/FLAIR sequences were associated with higher NfL levels at diagnosis in idiopathic/teratoma associated NMDAR-AE **(C)**, and majority of patients with MRI changes had elevated CSF-NfL levels at diagnosis **(D)**. *p value < 0.05, **p value < 0.01.

**Figure 5:**
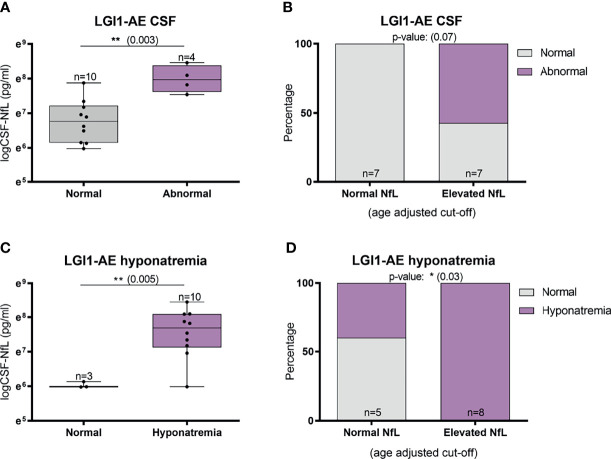
CSF-Neurofilament light chain (NfL) levels and abnormal CSF findings and hyponatremia in LGI1-AE patients. In LGI1-AE patients, an abnormal CSF finding (pleocytosis, elevated protein or oligoclonal bands) was associated with higher NfL levels at diagnosis **(A)** and patients with normal NfL levels at diagnosis always had normal CSF findings **(B)**. In addition, higher NfL levels at diagnosis was associated with the presence of hyponatremia **(C)**, and all patients with elevated NfL levels presented hyponatremia during the acute phase **(D)**. *p value < 0.05, **p value < 0.01.

Abnormal involuntary movements and abnormal brain MRI were associated with higher CSF-NfL levels in idiopathic/teratoma associated NMDAR-AE patients (p=0.002 and p=0.009, respectively) (**Figure 4A, C**). All patients with elevated CSF-NfL levels presented abnormal movements (p=0.05) (**Figure 4B**). In LGI1-AE, CSF-NfL levels were higher in patients with an abnormal CSF (p=0.003) and hyponatremia (p=0.005) (**Figure 5A, C**). Patients with normal CSF findings always had normal NfL levels (**Figure 5B**) and all patients with elevated CSF-NfL levels presented hyponatremia (p=0.03) (**Figure 5D**). No association between CSF-NfL levels and seizures, abnormal CSF or intensive care unit (ICU) admission in NMDAR-AE patients, or with abnormal movements or MRI findings in LGI1-AE patients was found (data not shown).

### Treatment Regimen and Follow-Up CSF-NfL Measurements

**Figure 6:**
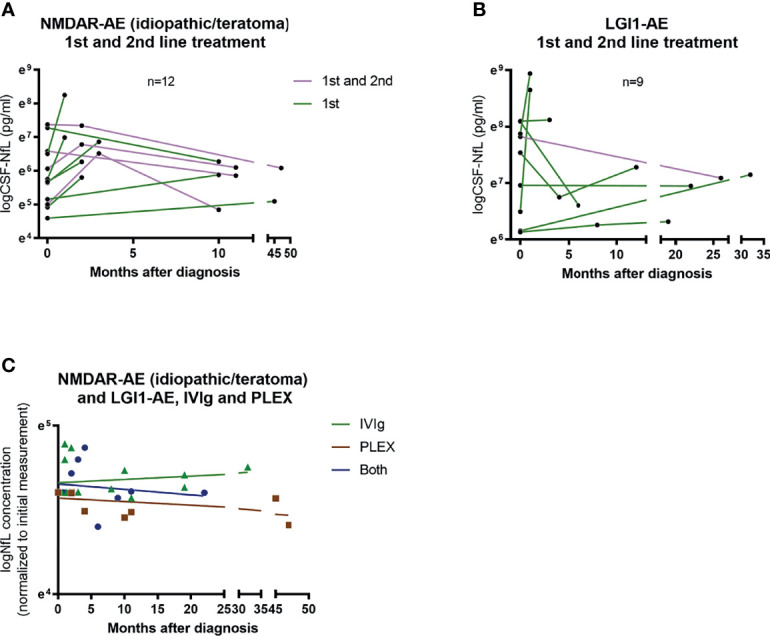
CSF-NfL levels and treatment regimens. Overview of patients (idiopathic/teratoma associated NMDAR-AE n=12 and LGI1-AE n=9) with available follow-up CSF-NfL measurements and if they received first or second line therapy **(A, B)**. A comparison between follow-up CSF-NfL values and different first line treatment strategies showed a tendency towards lower follow-up CSF-NfL values in patients treated with PLEX or a combination of PLEX/IVIg **(C)**. Patients only receiving IVIg are shown as green ▲. Patients only receiving PLEX are shown as brown ▪. And patients receiving both as blue ●.

Of the 12 NMDAR-AE patients with follow-up CSF-NfL measurements, eight received first-line treatment and four required additional second-line treatment (**Figure 6A**). In the nine LGI1-AE patients, eight received first-line and only one second-line treatment (**Figure 6B**). Plasma exchange (PLEX) or a combination of PLEX and intravenous immunoglobin (IVIg) seemed to reduce NfL levels more than IVIg alone (**Figure 6C**). But due to small sample size and unequal follow-up times these results must be interpreted with caution.

### Multivariable Analysis of Factors Influencing Poor Outcome and Elevated NfL at Diagnosis

Testing some of the single variables investigated above in a multivariable logistic regression model (parameters included: age, follow-up time and CSF-NfL level at diagnosis) similarly revealed that higher CSF-NfL at diagnosis was associated with a poorer outcome in idiopathic/teratoma associated NMDAR-AE and LGI1-AE patients combined (OR 11.97, 95% CI 1.22-117.01, p=0.033) (**Table 2**). When evaluating factors influencing NfL elevation (parameters included: age, pleocytosis and abnormal MRI), abnormal MRI was associated with an elevated NfL level at diagnosis (OR 5.4, 95% CI 1.05-27.88, p=0.044).

**Table 2:** Multivariable analysis examining predictors for poor outcome (mRS >2).

y (poor outcome)	Odds ratio	Std. error	p-value	[95% conf. interval]
**logNfL**	11.97205	13.92513	0.033	.224911 - 117.0125
**Age**	1.040413	.0364072	0.258	.9714481 - 1.114274
**Follow-up time**	.9999705	.0006332	0.963	.9987301 - 1.001212

Number of observations = 37 (idiopathic/teratoma associated NMDAR-AE and LGI1-AE patients).

## Discussion

We examined the relation between CSF-NfL levels and neuronal surface autoantibody titers, clinical presentation, ancillary findings and long-term outcomes in a national AE cohort, including 74 CSF samples from 53 patients. We found differences in CSF-NfL levels not only between NMDAR- and LGI-AE patients, but also between different NMDAR-AE etiologies. NfL levels at diagnosis appeared to be associated with long-term outcomes in both our univariable and multivariable analysis. This is in line with not only previous findings in other inflammatory diseases, but also a smaller study in AE (22, 24, 31). Five previous smaller studies on NfL as a biomarker in AE have been published (31–36).

We evaluated NMDAR-AE patients based on underlying etiology and found that gender distribution, median age and outcomes were widely different between idiopathic NMDAR-AE or NMDAR-AE secondary to HSE or concomitant with malignancies or demyelinating disease. Teratoma patients were grouped with idiopathic NMDAR-AE, as they turned out to present a similar clinical phenotype and disease course, although a paraneoplastic phenomenon. In addition, our study revealed that CSF-NfL levels were lower at diagnosis in idiopathic NMDAR-AE (teratomas included) when compared to secondary NMDAR-AE forms.

Higher CSF-NfL values in NMDAR-AE patients reported by previous studies may reflect combined evaluation of all NMDAR-AE etiologies (31, 32, 34). The CSF-NfL variations seen amongst different NMDAR-AE etiologies in this study, may suggest that further investigations towards underlying concomitant diseases such as malignant or demyelinating conditions might be considered in cases with high NfL levels. This does, however, not apply for teratoma-induced NMDAR-AE as only one of three teratoma patients presented slightly elevated CSF-NfL at diagnosis.

A recent study reported lower CSF-NfL values in patients with ovarian teratoma (36). In line with this, the two of our three teratoma patients had NfL levels below the median of the remaining idiopathic NMDAR-AE cohort. Teratoma patients have been shown to have a noticeably peripheral generation of autoantibodies, and have often been reported with higher NMDAR IgG levels in serum than CSF. This pronounced peripheral antibody production, might explain lower CSF-NfL levels in teratoma patients. Nevertheless, our three teratoma patients all presented more strongly positive assay readouts in CSF than in serum (+++ vs ++/+/-), not completely compiling with previous results published (37). Why our patients differ remains unknown.

Our study suggests that idiopathic and teratoma induced NMDAR-AE patients have lower CSF-NfL levels than patients with LGI1-AE at disease onset. This is in accordance with two previous studies and could be expected due to age differences between the diseases (32, 33). Nevertheless, the difference remained solid when adjusting CSF-NfL levels to age adjusted cut-off values when performing univariable analysis and when we corrected for age in multivariable analysis. In contrast, a recent study reported higher CSF-NfL levels in NMDAR-AE patients (36). However, that study investigated only 11 NMDAR and 10 LGI1/CASPR2-AE patients, did not consider other underlying disease etiologies and used an Enzyme-Linked Immunosorbent Assay (ELISA) based test. Ideally, the factors responsible for the difference in CSF-NfL levels between NMDAR- and LGI1-AE should be investigated by multivariable analysis, but this was not possible due to small sample size in previous and this present study.

It has been shown that intrathecal LGI1-IgG synthesis correlates with worse outcome, suggestive of greater neuronal injury in these patients (38). LGI1-AE has an insidious onset, with an often milder disease course than NMDAR-AE. Nevertheless, LGI1-AE patients more often present MRI changes and temporal lobe atrophy at follow-up, suggesting a more pronounced neurodegenerative pathophysiology. MRI findings such as white matter changes, likely result in greater neuroaxonal injury, and may consequently contribute to the higher CSF-NfL levels found in LGI1-AE patients. In line with this, our NMDAR-AE patients with abnormal MRI findings showed higher CSF-NfL levels than patients with a normal MRI. NMDAR-AE patients with MRI abnormalities and abnormal movements, may represent a fraction of patients with greater neuronal damage, and thus higher CSF-NfL values compared to patients without these features. Logistic regression, even though restricted by sample size, also suggested abnormal MRI as a factor predicting elevated CSF-NfL at diagnosis.

Follow-up measurements in our AE cohort indicated steadily decreasing levels in NMDAR-AE and a more heterogenous pattern in LGI1-AE patients. This might reflect a subtle, but ongoing, neurodegeneration in LGI1-AE patients, even during recovery. A recent study reported persisting long-term cognitive impairment in LGI1-AE, but whether these are temporarily progressing is unknown (39). The one patient in our study who had LGI1-AE without cognitive impairment, had the lowest CSF-NfL concentration at diagnosis, speculating that lower CSF-NfL levels might reflect lower neurodegeneration and thus no cognitive decline.

LGI1-AE patients with abnormal CSF findings and with hyponatremia had the highest CSF-NfL concentrations. This may reflect, that patients with CSF abnormalities (normally infrequent in LGI1-AE) are more severely affected by inflammation. The mechanisms behind the hyponatremia in LGI1-AE is speculated to be caused by hypothalamic neuronal impairment or LGI1 channel dysregulation in the renal epithelium. Higher CSF-NfL levels in patients with hyponatremia may reflect a more widespread disease, affecting the brain more globally and with a greater systemic affection compared to patients without hyponatremia.

We found no connection between elevated CSF-NfL levels and seizure activity or ICU admission. To our knowledge, scarce publications on seizures/epilepsy and serum NfL levels exist, that show none or sparse temporal elevation of NfL levels after seizure activity (40).

When comparing studies on NfL, the method used for evaluation is of essence. We used Simoa technology which has a higher sensitivity than ELISA and Electrochemiluminescence assays (41). Thus, Simoa has been reported to be 126 times more sensitive than ELISA, with an analytical sensitivity of 0.62 pg/mL compared to up to 78 pg/mL in ELISA (41). In addition, the test material has an impact on assay performance, as CSF displays higher sensitivity and specificity than both plasma and serum (23, 27, 42). Therefore, comparability of NfL concentrations between studies using different assay platforms and different test material is limited.

Reference values and clinical validated cut-off values (decision limits) for NfL levels are still lacking. Further studies on paired CSF and serum samples, including healthy controls, would be beneficial to enlighten NFL’s utility as a disease monitoring entity.

The strength of our study is the national NMDAR- and LGI1-AE cohort database used to provide accurate clinical and paraclinical information, coupled with a long follow-up time and sensitive CSF-NfL testing on Simoa. This allowed us to test a substantial number of patients.

Limitations include the small sample size and the retrospective approach, which constrained statistical analysis. However, our main aim was to evaluate CSF-NfL levels at time of diagnosis and relate to long-term outcomes. Performing exploratory univariable analysis, as we do in this study, composes a risk of overinterpretation and overlooking potential confounders. To accommodate this, we performed multivariable testing. However, due to the sample size, the model could only fit three predictors, and unfortunately did not allow to adjust for antibody subtype (NMDAR or LGI1) or treatment regimen (secondline), which are two important variables (4, 43, 44). Thus, our conclusions should be interpreted with caution.

Using the mRS to evaluate long-term outcome of AE patients comprises a restriction, as recent studies have shown that AE patients often suffer from cognitive impairment even years after treatment (45, 46). Consequently, other outcome-evaluating tools with greater focus on cognitive function and quality of life are warranted.

In conclusion, CSF-NfL levels at diagnosis were higher in LGI1-AE patients than in idiopathic and teratoma associated NMDAR-AE and our data indicate that high CSF-NfL may relate to worse long-term outcome. Furthermore, NfL levels might help distinguish idiopathic/teratoma associated NMDAR-AE from secondary NMDAR-AE forms. Additional studies on larger cohorts, with standardized methods and cut-off values are warranted to further validate CSF-NfL as a potential biomarker in AE.

## Data Availability Statement

The raw data supporting the conclusions of this article will be made available by the authors, without undue reservation.

## Ethics Statement

The studies involving human participants were reviewed and approved by Danish Medical Authorities (3-3013-3214/1), the Ethical Committee of the Region of Southern Denmark (nr. S-20180026) and the Danish Data Protection Agency. Written informed consent from the participants’ legal guardian/next of kin was not required to participate in this study in accordance with the national legislation and the institutional requirements.

## Author Contributions

MN and MB, drafting/revision of the manuscript for content, including medical writing for content, major role in the acquisition of data, study concept or design, and analysis or interpretation of data. MR, JM, and DO, drafting/revision of the manuscript for content, including medical writing for content, and analysis or interpretation of data. UH, major role in analysis and interpretation of data. AN and ML, drafting/revision of the manuscript for content, including medical writing for content, and major role in the acquisition of data. ZI, drafting/revision of the manuscript for content, including medical writing for content, study concept or design, and analysis or interpretation of data. All authors contributed to the article and approved the submitted version.

## Funding

MN received funding from Odense University Hospital and the University of Southern Denmark.

## Conflict of Interest

The authors declare that the research was conducted in the absence of any commercial or financial relationships that could be construed as a potential conflict of interest.

## Publisher’s Note

All claims expressed in this article are solely those of the authors and do not necessarily represent those of their affiliated organizations, or those of the publisher, the editors and the reviewers. Any product that may be evaluated in this article, or claim that may be made by its manufacturer, is not guaranteed or endorsed by the publisher.

## Abbreviations

AE Autoimmune Encephalitis; CBA Cell-Based Assay; ELISA Enzyme-Linked Immunosorbent Assay; FBDS Faciobrachial Dystonic Seizures; HSE Herpes Simplex Virus type 1 Encephalitis; ICU Intensive Care Unit; IgG1 Immunoglobulin G1; IgG4 Immunoglobulin G4; IVIg Intravenous Immunoglobulin; LE Limbic Encephalitis; LGI1 Leucine-rich Glioma-Inactivated 1; mRS Modified Rankin Scale; NfL Neurofilament Light Chain; NMDAR *N*-methyl-D-Aspartate receptor; OCB Oligoclonal Bands; PLEX Plasma Exchange; Simoa Single Molecule Array.
