# Supplementary figures and images for: CSF-Neurofilament Light Chain Levels in NMDAR and LGI1 Encephalitis: A National Cohort Study
Source: Front Immunol. 2021 Dec 16;12:719432. doi: 10.3389/fimmu.2021.719432 (PMC8716734; doi:10.3389/fimmu.2021.719432)

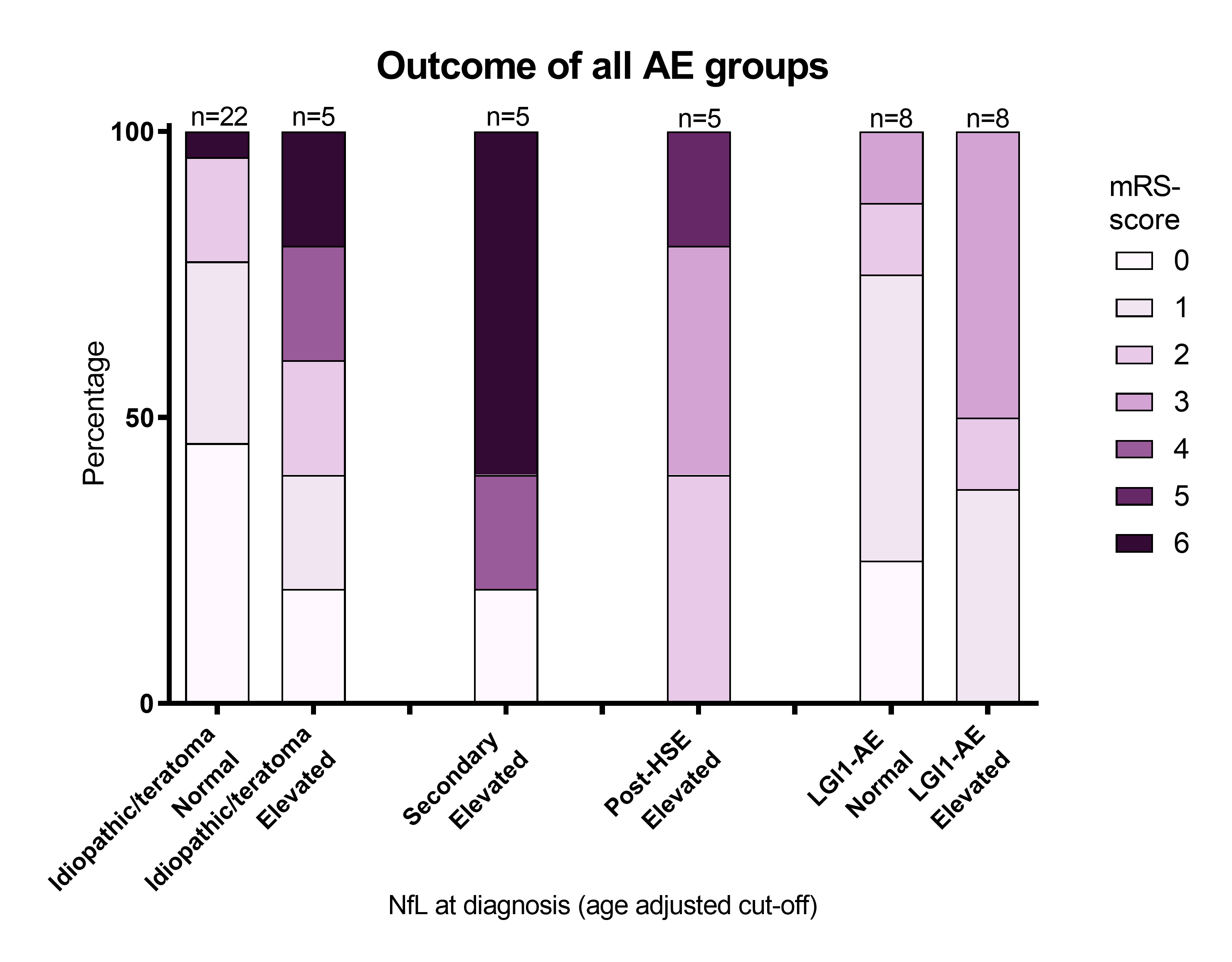

Supplement: Supplementary file 1 [file Image_1.tif]
