# Supplementary material for: CSF-Neurofilament Light Chain Levels in NMDAR and LGI1 Encephalitis: A National Cohort Study
Source: Front Immunol. 2021 Dec 16;12:719432. doi: 10.3389/fimmu.2021.719432 (PMC8716734; doi:10.3389/fimmu.2021.719432)
Supplement: Supplementary file 2 [file Table_1.docx]

**Supplementary Table 1. Overview of individual AE patients included in the study**

| Patient ID | Age | Sex | NMDAR IgG | | IgG titer  at diagnosis | | CSF-NfL prior to diagnosis | | | CSF-NfL at diagnosis | | CSF-NfL at follow-up | | Poor outcome at last follow-up | | Final diagnosis | |  |
| --- | --- | --- | --- | --- | --- | --- | --- | --- | --- | --- | --- | --- | --- | --- | --- | --- | --- | --- |
| **Primary NMDAR-AE** | Years | F/M | Ab presence | | CSF/serum | | | pg/mL  (months after  diagnosis) | | pg/mL  (months after  diagnosis) | | pg/mL  (months after  diagnosis) | | mRS>2 | | Given at the hospital | |  |
| 2 | 11 | F | CSF+S | | (+++/++) | | |  | | 492 (0) | | NA | | no | | NMDAR-AE | |  |
| 3 | 52 | F | CSF+S | | (+++/++) | | |  | | 667 (0) | | 3821 (1) | | no | | NMDAR-AE | |  |
| 4 | 15 | F | CSF+S | | (++/++) | | |  | | 99 (-1) | | 163 (45) | | no | | NMDAR-AE | |  |
| 5 | 36 | M | CSF/no S | | (+++/-) | | |  | | 569 (0) | | NA | | no | | NMDAR-AE | |  |
| 6 | 35 | M | CSF+S | | (+++/++) | | |  | | 1598 (-1) | | 1545 (3), 439 (47) | | yes | | NMDAR-AE | |  |
| 9 | 21 | F | CSF+S | | (+/+) | | |  | | 293 (-1) | | 526 (3) | | no | | NMDAR-AE | |  |
| 13 | 34 | M | CSF/neg. S | | (++/-) | | |  | | 315 (0) | | 1082 (1) | | no | | NMDAR-AE | |  |
| 14 | 19 | M | CSF/neg. S | | (++/-) | | |  | | 174 (0) | | 357 (22) | | no | | NMDAR-AE | |  |
| 17 | 19 | F | CSF+S | | (+++/+) | | |  | | 429 (0) | | 883 (2) - 445 (11) | | no | | NMDAR-AE | |  |
| 24 | 17 | F | CSF/neg. S | | (+++/-) | | |  | | 98 (0) | | NA | | no | | NMDAR-AE | |  |
| 25 | 55 | F | CSF+S | | (+++/++) | | |  | | 1812 (0) | | NA | | death | | NMDAR-AE | |  |
| 26 | 15 | F | CSF+S | | (+++/++) | | |  | | 169 (0) | | NA | | no | | NMDAR-AE  (teratoma) | |  |
| 27 | 16 | F | CSF/no S | | (++/-) | | |  | | 129 (0) | | NA | | no | | NMDAR-AE | |  |
| 29 | 28 | F | CSF+S | | (+++/NA) | | |  | | 1447 (0) | | 531 (10) | | no | | NMDAR-AE | |  |
| 31 | 24 | F | CSF+S | | (+++/+) | | |  | | 149 (0) | | 676 (3) -127 (10) | | no | | NMDAR-AE | |  |
| 35 | 42 | M | CSF/neg. S | | (+++/-) | | |  | | 284 (0) | | 955 (3) | | no | | NMDAR-AE | |  |
| 36 | 18 | M | CSF+S | | (+++/++) | | |  | | 408 (0) | | NA | | no | | NMDAR-AE | |  |
| 38 | 22 | F | CSF/neg. S | | (++/-) | | |  | | 175 (0) | | NA | | no | | NMDAR-AE | |  |
| 39 | 65 | M | CSF+S | | (++/+) | | |  | | 1588 (0) | | NA | | death | | NMDAR-AE | |  |
| 40 | 33 | F | CSF/neg. S | | (+/-) | | |  | | 175 (-1) | | NA | | no | | NMDAR-AE | |  |
| 50 | 18 | F | CSF+S | | (+++/+) | | |  | | 723 (0) | | 350 (11) | | no | | NMDAR-AE  (teratoma) | |  |
| 56 | 27 | F | CSF/neg. S | | (+++/-) | | |  | | 137 (0) | | 332 (2) | | no | | NMDAR-AE | |  |
| 57 | 18 | F | CSF/neg. S | | (+++/-) | | |  | | 339 (0) | | NA | | no | | NMDAR-AE | |  |
| 62 | 23 | F | CSF+S | | (++/+) | | | 106 (-3) | | 256 (0) | | NA | | no | | NMDAR-AE | |  |
| 67 | 13 | F | CSF/no S | | (+++/-) | | |  | | 34 (-) | | NA | | no | | NMDAR-AE  (teratoma) | |  |
| 60 | 20 | F | CSF/no S | | (++/-) | | |  | | 172 (0) | | NA | | no | | NMDAR-AE | |  |
| 69 | 15 | F | CSF+S | | (+++/NA) | | |  | | 151 (-) | | NA | | no | | NMDAR-AE | |  |
| **Secondary post-HSE NMDAR-AE** | | |  | |  | | |  | |  | |  | |  | |  | |  |
| 12 | 49 | M | CSF/neg. S | | (++/-) | | |  | | 4898 (0) | | 4767 (1) | | no | | post-HSE NMDAR-AE | |  |
| 16 | 70 | M | CSF+S | | (+++/+) | | | 1352 (-5) | | 14475 (0) | | NA | | yes | | post-HSE NMDAR-AE | |  |
| 30 | 46 | M | CSF/no S | | (+++/-) | | | 9922 (-2) | | 12409 (0) | | NA | | no | | post-HSE NMDAR-AE | |  |
| 45 | 69 | F | CSF/neg. S | | (+++/-) | | |  | | 22458 (0) | | NA | | yes | | post-HSE NMDAR-AE | |  |
| 61 | 65 | F | CSF/no S | | (+/-) | | | 17600 (-9), 14652 (-8) | | 7115 (-2) ^a^ | | NA | | yes | | post-HSE NMDAR-AE | |  |
| **Secondary Other NMDAR-AE** | | |  |  | |  | | |  | |  | |  | |  | |  | |
| 10 | 56 | M | CSF+S | | (++/++) | | |  | | 1977 (-3) ^b^ | | 840 (7) | | death | | NMDAR-AE concomitant  with MS | |  |
| 21 | 74 | M | CSF+S | | (+++/+++) | | |  | | 1969 (-1) | | NA | | death | | NMDAR-AE concomitant with  paraneoplastic LE  with anti-Hu (SCLC) | |  |
| 22 | 67 | M | CSF+S | | (+++/++) | | | 5196 (-1) | | 8121 (0) | | NA | | death | | NMDAR-AE concomitant with  PCNSL (B-cell) | |  |
| 46 | 26 | M | CSF/neg. S | | (+/-) | | |  | | 2285 (0) | | 2876 (1) | | no | | NMDAR-AE concomitant with  ADEM ^c^ | |  |
| 48 | 32 | F | CSF/no S | | (+/-) | | |  | | 28048 (0) | | NA | | yes | | NMDAR-AE concomitant with  MS | |  |
| **LGI1-AE** | | | | | | | | | | | | | | | | | |  |
| 22 | 60 | F | CSF/S | | (+/+) | | |  | | 397 (2) | | NA | | no | | LGI1-AE with LE | |  |
| 23 | 60 | M | CSF/S | | (+/++) | | |  | | 659 (0) | | 5701 (48) | | no | | LGI1-AE with LE | |  |
| 7 | 57 | F | CSF/S | | (+/+) | | |  | | 1305 (0) | | 1899 (0) | | no | | LGI1-AE with LE | |  |
| 11 | 60 | M | CSF/S | | (+++/+) | | |  | | 3280 (-1) | | 3355 (4) | | no | | LGI1-AE with LE | |  |
| 14 | 82 | F | CSF neg/S | | (-/+++) | | |  | | 985 (-1) | | NA | | no | | LGI1-AE with LE | |  |
| 5 | 66 | M | CSF/S | | (+/+) | | | 755 (-11) | | 4722 (0) | | NA | | yes | | LGI1-AE with LE | |  |
| 3 | 71 | F | CSF/S | | (+/+++) | | |  | | 1052 (0) | | 1041 (22) | | no | | LGI1-AE with LE | |  |
| 17 | 61 | F | CSF/S | | (-/++) | | |  | | 470 (0) | | 1271 (43) | | no | | LGI1-AE with LE | |  |
| 19 | 47 | M | CSF/S | | (++/++) | | |  | | 460 (0) | | 522 (8), 554 (19) | | no | | LGI1-AE with LE | |  |
| 18 | 65 | M | CSF/S | | (++/+++) | | |  | | 3259 (-1) | | 739 (7) | | yes | | LGI1-AE with LE | |  |
| 9 | 67 | M | CSF/S | | (+/++) | | |  | | 1878 (-1) | | 853 (4), 1451 (12) | | yes | | LGI1-AE with LE | |  |
| 13 | 73 | M | CSF/S | | (+/NA) | | |  | | 750 (0) | | NA | | yes | | LGI1-AE with LE | |  |
| 10 | 68 | M | CSF NA/S | | (NA/++) | | |  | | 2490 (-1) | | 1200 (26) | | yes | | LGI1-AE with LE | |  |
| 8 | 32 | F | CSF/S | | (+/+++) | | |  | | 2628 (0) ^d^ | | 7626 (1) ^d^ | | no | | LGI1-AE with LE | |  |
| 6 | 30 | M | CSF/S | | ((+)/++) | | |  | | 1543 (0) | | NA | | no | | LGI1-AE with LE | |  |
| 2 | 80 | F | CSF/S | | (++/++) | | |  | | 395 (0) | | NA | | no | | LGI1-AE exclusively with  FBDS | |  |

^a^ This patient’s NfL sample was obtained 1,5 months prior to definite diagnosis (antibody confirmation), the patient showed clear clinical signs of NMDARE at the timepoint of the sample.

^b^ This patient’s NfL sample was obtained 2,5 months prior to definite diagnosis (antibody confirmation), the patient showed clear clinical signs of NMDARE at the timepoint of the sample.

^c^ MOG-antibody was not tested

^d^ This patient received initial treatment for AE at second NfL measurement, 1 month after diagnosis.Thus the follow-up NfL measurement is around time of treatment, and was not used in the study. .

Abbreviations: AE=Autoimmune Encephalitis; NMDAR= *N*-methyl-D-aspartate receptor; Post-HSE= Post Herpes Simplex Virus type 1 encephalitis; LGI1= Leucine-rich Glioma-Inactivated 1; CSF=cerebrospinal fluid; MRI; Magnetic Resonance Imaging; NfL=Neurofilament Light Chain; mRS=Modified Rankin scale; MS=Multiple Sclerosis; SCLC= Small-cell lung cancer; PCNSL= Primary CNS lymphoma; ADEM= Acute disseminated encephalomyelitis; LE= Limbic Encephalitis; FBDS= Faciobrachial Dystonic Seizures; NA=Not available
